# Effect of thyme, ginger, and their nano-particles on growth performance, carcass characteristics, meat quality and intestinal bacteriology of broiler chickens

**DOI:** 10.1186/s12917-024-04101-z

**Published:** 2024-06-22

**Authors:** Amal H. A. Hassan, Ibrahim M. I. Youssef, Nasser S. Abdel-Atty, Asmaa S. A. Abdel-Daim

**Affiliations:** 1 Department of Food Safety and Technology, Faculty of Veterinary Medicine, Beni-Suef University, Beni-Suef, 62511 Egypt; 2 Department of Nutrition and Clinical Nutrition, Faculty of Veterinary Medicine, Beni-Suef University, Beni-Suef, 62511 Egypt

**Keywords:** Thyme, Ginger, Nano-thyme, Nano-ginger, Performance, Meat quality, Broilers

## Abstract

This study was conducted to evaluate the effects of thyme, ginger, and their nano-particles, as alternatives to antibiotic growth promotors (AGP), on productive performance, carcass traits, meat quality and gut health of broiler chickens. A total of 270 one-day-old broiler chicks were randomly distributed into 6 groups, each consisting of 3 replicates (*n* = 15 chicks/replicate). The birds in group 1 were fed the control diet which contained neither antibiotic growth promotors nor phytogenic feed additives (PFA). Birds in group 2 were fed diets containing 0.05% of AGP (Bacitracin methylene disalicylate). Chicks in group 3 and 4 were fed diets supplemented with 1.0% of thyme and ginger, respectively, whereas birds in group 5 and 6 were offered diets including 0.10% of nano-thyme and nano-ginger, respectively. The experiment lasted for 35 days. It was found that thyme and ginger with their nano-products, like the antibiotic, improved the body weight, weight gain and feed conversion rate of birds. The effect of ginger and nano-ginger on body weight and weight gain was greater than other treatments. During the overall feeding period, the feed cost of production was the highest in antibiotic group, but was the lowest in ginger and nano-ginger treatments. There was no effect of dietary treatments on carcass yield or organs weight except bursa of Fabricius and abdominal fat. Thyme, ginger and their nano-composites increased the weight of bursa and reduced the abdominal fat amount. The phytogenic additives and their nano-particles improved the colour, water holding capacity, and flavor of meat. Moreover, these additives reduced the total intestinal bacterial count as well as the total aerobic mesophilic count of meat. The effect of PFA and their nano-particles on the bacterial count was similar to that of antibiotic. In conclusion, thyme and ginger with their nano- particles can be considered as promising agents in feeding of broilers to improve the growth performance, gut health and meat quality. Moreover, these additives can be used as alternatives to AGP to overcome its health hazards and the high cost. The nanotechnology of herbal plants enables them to be added in smaller amounts in poultry diets with producing the same effect of raw ingredients, and this could be due to the higher bioavailability.

## Introduction

Poultry meat is one of the most available and cheapest sources of high-quality protein for human feeding. Nonetheless, broiler production is unfortunately facing several infectious threats caused by different pathogenic bacteria. Thus, production of safe and healthy chicken needs appropriate microbial control for high quality meat production [1]. Excessive use of antibiotics, to control the microbial infection, resulted in hazardous consequences in the form of antibiotic bacterial resistance development and drug residues in meat [2]. Therefore, the use of antibiotics is being banned all over the world [3]. Nowadays, various phytogenic feed additives are encouraged in poultry feed industry as alternatives to antibiotic growth promoters [2]. Plants are a rich source of bioactive composites with diverse pharmacological and biological actions [4]. Ginger and thyme as natural growth enhancers can be used as potential alternatives for antibiotic growth promoters [5].

Thyme (*Thymus vulgaris*) is one of these alternative medicinal herbs and is recognized to improve the appetite and feed intake. Also, it increases the secretion of endogenous digestive enzymes and stimulates the immune status in poultry, owing to its content from the phenolic substances [6]. Thymol and carvacrol are the most important bioactive compounds in thyme, which are responsible for its pharmacological properties [7]. It is well known that thymol and carvacrol have not only an antioxidant action but also antibacterial, antiviral and aroma regulatory activities [7]. Some studies reported that supplementation of poultry diets with thyme improved the performance indices [8, 9], however, other studies suggested that thyme had no effect [10].

Ginger (*Zingiber officinale)* is commonly used as a food spice and as a medicinal herb [11]. The main important composites in ginger are gingerol, gingerdiol and gingerdione, which have the ability to improve the digestive enzymes secretion, and decrease the microbial activity [12]. Therefore, it can be used to improve the health status and growth performance in poultry.

Nanotechnology is a promising and developing technology that has great potential to alter the agriculture and livestock sector all over the world. Nanotechnology procedure deals with the conversion of larger molecules to nanometer size [13]. The method of converting these larger molecules to small one causes alterations in the innate physical and chemical nature of the basal material. This technology produced products of special properties such as higher penetrability, reactivity, surface area and quantum activities which appear to be needed in various fields including animal nutrition, diagnosis and treatment of diseases, food safety, biosensors and etc… [14]. This novel strategy can be exploited in livestock and poultry feeding for efficient utilization of nutrients and other supplements, and better uptake of feedstuffs. Nevertheless, the technology holds greater potentials for better livestock and poultry production, studies are much limited. Nano additives are likely to have the benefit of better bioavailability, small dose rate and stable interaction with other compounds. Because of their low dose usage, they can be used as alternatives for antibiotics as growth promoters, remove antibiotic residues in the animal outputs, decrease the environmental pollution and produce hazards-free animal products [15].

Therefore, this study was carried out to investigate the effect of some phytogenic feed additives (thyme and ginger) and their nano-particle preparations in comparison with antibiotic on productive performance, gut health, carcass characteristics and meat quality of broilers. In addition, diets cost and its economic efficiency on meat production was calculated.

## Materials and methods

### Preparation and characterization of nano-products

Ginger *(Zingiber officinale*) and thyme (*Thymus vulgaris*) were bought from a local market, washed several times with water, cut into small pieces, and then air dried in shaded area. The dried plants were ground by a mixer to get fine powder which was then stored in a sterilized glass container at room temperature until to be used [16]. For the preparation of nanoparticles, the plant powder was milled by mechanical ball milling utilizing a planetary ball mill (Retsch PM 400, Germany) for 48 h. The central occurrence in mechanical milling is the ball–powder–ball collision, where the dried powder was trapped between the colliding balls during milling with high speed producing fine powder in nano scales.

The morphological characteristics of thyme and ginger nanoparticles were studied using the field transmission scanning electron microscope (FESEM, JEOL JSM-5900, Japan). A very thin layer of gold was first applied to the samples using the direct sputtering technique. The chemical structures of thyme and ginger nanoparticles were checked by using FTIR (Fourier Transform Infrared) spectroscopy analysis. The FTIR spectra were measured using a Bruker-Vertex 70 over the range of 4000–500 cm^− 1^.

### Birds and diets

The study was carried out on 270 one-day-old broiler chicks (Ross-308). The birds were obtained from a commercial hatchery, weighed and distributed, at random, into 6 groups. Each treatment included 3 replicates with 15 chicks per replicate. Chicks were housed in 3-tier battery in an equipped closed farm. The chicks of each replicate were set in one wire cage (1.5 m length × 1 m width × 0.5 m height) supplied with nipple drinkers, feeders and slatted iron floor. Feed and water were provided all time. The birds in group 1 were fed the control diet which contained neither antibiotic growth promotors (AGP) nor phytogenic feed additives (PFA). Birds in group 2 were fed diets containing 0.05% of AGP (Bacitracin methylene disalicylate) Chicks in group 3 and 4 were fed diets supplemented with 1.0% of thyme and ginger, respectively, whereas birds in group 5 and 6 were offered diets including 0.10% of nano-thyme and nano-ginger, respectively. The experiment lasted for 35 days. The diets were formulated to satisfy the nutrient requirements of broilers according to the nutritional specifications guide of the breed. Thyme and ginger were analyzed for the nutrient contents using AOAC [17] procedures. It was found that thyme contained 9.57% crude protein (CP), 6.07% ether extract (EE), 17.03% crude fiber (CF), 11.2% ash, 45.17% nitrogen free extract (NFE), and 2449.3 kcal ME/kg, whereas the ginger consisted of 8.81% CP, 5.22% EE, 15.67% CF, 4.96% ash, 56.31 NFE, and 2742.6 kcal ME/kg. The metabolizable energy content of thyme and ginger estimated from their proximate composition according to Pauzenga [18]. The ingredient and chemical compositions of the diets is shown in Table 1. The used dietary supplements were added to the basal diets substituting equal amounts of yellow corn. The feeding period was divided into two phases, in which starter (0–21 day) and grower (22–35 day) diets were fed. The ambient temperature was gradually reduced from 33°Cat day 1 of age to about 24 °C at the end of the experiment. The level of relative humidity was 70% during the brooding period and maintained between 50 and 70% throughout the experiment. The light during the first 4 days of age was provided for 24 h continuously. From day 5 of age onwards, the daily light was reduced to 18 h. Birds were allowed to have free access to water and feed along the experimental period.

**Table 1:** Physical and chemical compositions (%) of the basal diets (as fed basis) ^a^

Ingredient	Diet	
	Starter (0–21 d)	Grower (22–35 d)
**Physical composition**		
Yellow corn, ground	53.02	57.35
Soybean meal, 46% CP	37.00	30.62
Corn gluten, 60% CP	3.20	5.0
Vegetable oil^b^	2.40	3.0
Monocalcium phosphate	1.67	1.50
Limestone, ground	1.62	1.49
Common Salt	0.35	0.35
Minerals and vitamins premix^c^	0.30	0.30
L- Lysine HCL	0.22	0.23
DL-Methionine	0.17	0.13
Threonine	0.05	0.03
**Chemical composition (calculated)**		
Metabolizable energy, kcal /kg	3002.0	3109.4
Dry matter	90.83	90.92
Crude protein	23.02	21.50
Methionine	0.56	0.51
Lysine	1.44	1.29
Threonine	0.97	0.88
Crude fiber	2.65	2.52
Calcium	0.96	0.87
Phosphorus, available	0.48	0.44
Sodium	0.16	0.16

^a^Antibiotic, thyme, ginger, nano- thyme and nano-ginger were added to the diets of groups 2, 3, 4, 5 and 6 at the rate of 0.05, 1.00, 1.00, 0.1, and 0.1%, respectively, replacing equal amounts of yellow corn

^b^Vegetable oil composed of 75% sunflower oil and 25% of soybean oil

^c^Poultry mineral and vitamin premix “Avimix, Agri – Vet Company, Egypt“: each 3kg contain Vit.A, 12,000,000 IU; Vit.D_3_, 2,000,000 IU; Vit.E, 10,000 mg; Vit.K._3_, 2000 mg; Vit.B_1_, 1000 mg, Vit.B_2_, 5000 mg ; Vit.B_6_, 1500 mg; Vit. B_12_, 10 mg; biotin, 50 mg; pantothenic acid, 10000 mg; nicotinic acid, 30000 mg; folic acid,1000 mg, choline chloride, 250000 mg; Mn, 60000 mg; Zn, 50000 mg; Fe, 30000 mg; Cu, 10000 mg; I, 1000 mg; Se, 100 mg; Co, 100 mg; and calcium carbonate up to 3kg

### Growth performance traits

Feed was offered to the birds daily, and the feed intake per day was calculated after removal of the refused feed. The chicks were weighed weekly, and consequently the weekly weight gain was measured. Based on the feed intake and weight gain, the feed conversion rate was estimated. The feed conversion ratio (FCR) was corrected for dead birds. The mortality rate was recorded daily throughout the experiment.

### Carcass characteristics

At the end of each experiment, 6 birds per treatment (two from each replicate) were randomly selected and weighed live, sacrificed by neck cut and allowed to bleed. Then, the chickens were scalded, de-feathered and carcasses eviscerated. The gizzard, heart, liver, were excised and weighed. The weight of carcass refers to the weight of the eviscerated carcass plus giblets (liver, heart, skinned empty gizzard). Carcass yield (dressing percentage) was obtained by expressing the dressed carcass weight (with giblets) as a percentage of the live body weight. Abdominal fat was separately recorded for each bird and expressed as a percentage of the live body weight.

### Meat quality parameters

Samples of broiler cuts (breast and thigh muscles) were taken from six slaughtered birds of each treatment at the end of experiment, and the physico-chemical and sensory characteristics of broiler cuts were determined as follow:

#### pH values

The pH of broiler chicken cuts was measured at 2 and 24 h post mortem according to AOAC [17], and it was determined using a pH meter.

#### Water holding capacity

The water holding capacity of breast and thigh muscles were determined according to Hamm [19]. The obtained results were expressed as a percentage of exuded water in relation to the starting sample weight.

#### Shear force

The broiler chicken breast samples of each treated group were oven cooked at 180 ^o^C for 20 min to attain an internal temperature of 70^o^C. Samples were left to cool at room temperature then used for the tenderness. The shear force (kgf/cm^3^) was then determined using Instron Universal Testing Machine (Model 2519 − 105, USA) at crosshead speed of 200 mm/min. Six tests from each sample were taken. The results were expressed as kilogram force (kg f) to shear.

#### Instrumental color measurements

Instrumental color determination was made on the surfaces of skinless breast samples using Chroma meter (Konica Minolta, model CR 410, Japan) calibrated with a white plate and light trap supplied by the manufacturer. Color was expressed using the standard CIE LAB color system as follows: a-value (redness/green), b-value (yellowness/blue) and L-value (lightness/darkness,). Three readings were taken at various points of each breast sample [20].

#### Chemical composition

Six broiler chickens, with a body weight close to the overall mean, from each treatment were chosen. The chickens were weighed after being exposed to 24 h -feed fasting with free access to water, and slaughtered by neck dislocation. The birds were scalded, defeathered, and eviscerated after removal of head, neck and legs. The breast and thigh muscles of each bird were separated from the carcass, dissected into small pieces, and then dried, individually, in a hot air oven at 70 °C for 48 h. After reaching a constant weight, the muscles were weighed and its DM was calculated. Thereafter, the meat muscles were ground by using an electrical grinder, homogenized, and analyzed for CP, EE, and ash according to AOAC [17] methods.

#### Sensory analysis

Sensory characteristics of raw and cooked broiler chicken cuts were carried out immediately after slaughtering by fifteen pre-trained panelists from Food Safety and Technology Department, Beni-Suef University, Egypt. Broiler chicken cuts were cooked in a forced draught oven (Heraeus D-63,450 Hanau, Germany) at 180 ºC to core temperature of 75 ºC. Sensory characteristics in the term of appearance, flavor/odor, tenderness and overall acceptability of the cooked samples were determined using a nine-point hedonic scale (0–9). The scale points were: excellent, 9; very good, 8; good, 7; acceptable, 6; poor below 6; a score of 6 was taken as the lower limit of acceptability [21].

### Bacteriological examination

#### Determination of total aerobic mesophilic (TAM) count in meat

At the end of experiment, broiler cuts (breast and thigh muscles) of six birds per treatment were used for determination of total aerobic mesophilic count. About 10.0 g of each sample were homogenized (Lab Blender 400; Seward Medical Ltd., London, UK) with 90 ml of 0.1% sterile peptone water (Merck, Darmstadt, Germany) for 2 min and series of dilutions up to 10^− 7^ were prepared. Standard plate count agar (6G2307, Biolife, Italy) was used for enumeration of total aerobic mesophilic bacteria ISO [22]. The inoculated plates were incubated at 37 °C for 48 h. The plates that contained colonies between 30 and 300 were counted; the average of the count was multiplied by the dilution factor to get the total mesophilic count per gram of flesh. Microbiological data were transformed into logarithms of the number of colony forming units (CFU/g).

#### Determination of intestinal bacterial count

At the end of experiment, the fresh digesta samples from the caeca of 6 birds per treatment were individually collected directly after slaughter in separate sterile Petri dishes. About 1.0 g of caeca content was transferred into 9 mL peptone water. The suspension was homogenized for 2 min and serially diluted up to 10^− 7^. Violet red bile glucose agar (CM 485, Oxoid, UK) was used for enumeration of total *Enterobacteriaceae* count ISO [23]. The inoculated plates were incubated at 37 °C for 48 h. Moreover, 1 ml of intestinal dilution was inoculated into three replicate tubes of Lauryl Sulphate Tryptose Broth (LST, Oxoid, CM451) with inverted Durham’s tubes and incubated at 35^◦^C for 48 h for determination of the most probable number of coliforms. Microbiological data were transformed into logarithms of the number of colony forming units (CFU/g).

### Economic efficiency measurement

Economic evaluation of the experimental diets was performed by calculating the cost of feed per kilogram, cost of total feed intake and feed cost per kilogram weight gain. To obtain the cost of each kg weight gain produced, the cost of the consumed feed amounts in each treatment was calculated and divided by the weight gain of the birds, or the price of each kg feed is multiplied by the feed conversion rate.

### Statistical analyses

The results were analyzed statistically using SPSS statistical program (IBM, version 20, Chicago, USA, 2011). The data were subjected to one-way ANOVA accompanied by Duncan’s multiple range tests to detect the differences between the treatments. Pearson Chi square test was used for evaluating the mortality percentages. Moreover, the sensory characteristics score of meat was analyzed by using non-parametric tests; therefore, Kruskal-Wallis-test was used to examine the significant differences between means. The results were presented as means ± SEM. Probability values less than 0.05 (*P* < 0.05) was considered significant.

## Results

**Fig. 1:**
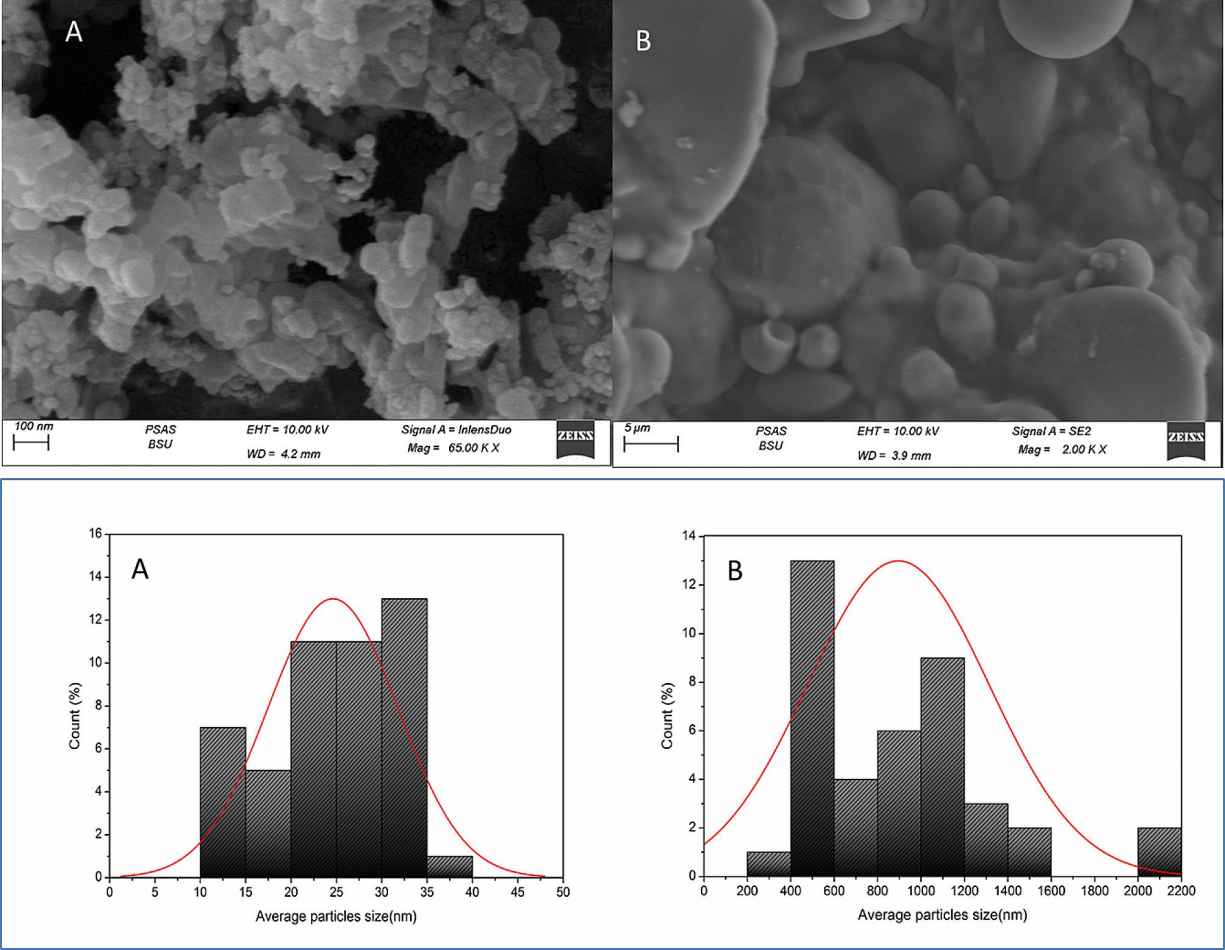
SEM (Scanning electron microscope) images and particles size distribution of nano-ginger (**A**) and nano-thyme (**B**)

The characteristic morphological features of milled thyme and ginger are illustrated in Fig. 1. The milled ginger appears as spherical agglomerated particles with nano-scale size (24.6 nm ± 1.03). However, milling of thyme resulted in spherical particles with a larger size (895 nm ± 66).

**Fig. 2:**
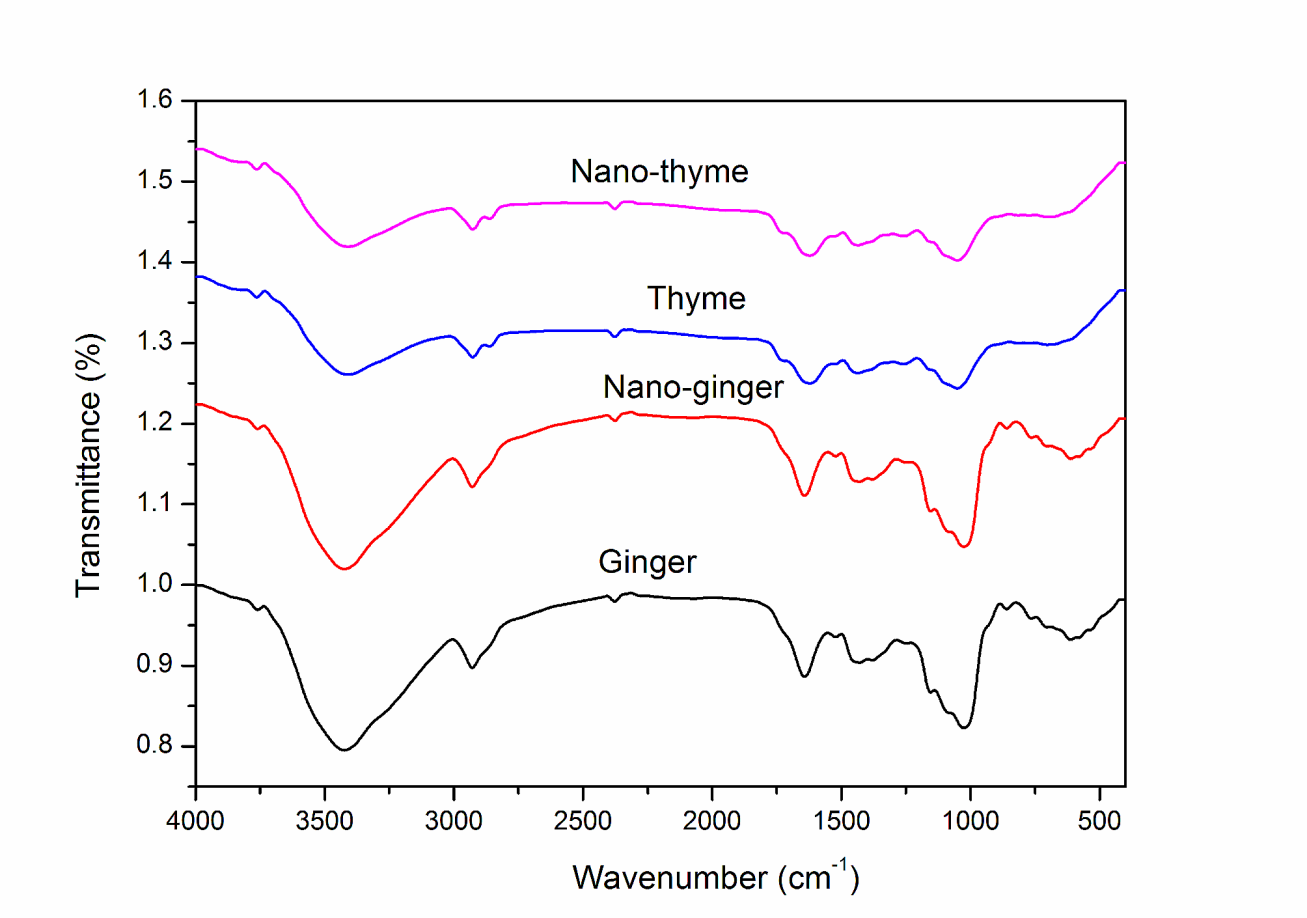
FTIR (Fourier Transform Infrared) spectra of prepared nano-thyme and nano-ginger powder

The functional groups of milled thyme and ginger were measured by FTIR (Fig. 2). The FTIR spectra of the ball-milled thyme and ginger were found at wavenumber between 500 and 4000 cm^− 1^ at room temperature. A wide absorption peak at 3410, 3423 cm-1 corresponds to -OH stretching vibrations of water molecules. The bands show the functional groups of phytochemicals found in the examined materials are detailed in Table 2. The results demonstrated that the ball milling produced no new chemical group and no changes in transmittance and or wavenumber.

**Table 2:** FTIR data of prepared nano-thyme and nano-ginger powder

FTIR spectra of nano- thyme		FTIR spectra of nano-ginger	
Wavenumber cm^− 1^	Functional group	Wavenumber cm^− 1^	Functional group
3410	O-H	3423	O-H
2926	C-H	2928	C-H
2800	C-H	1642	H_2_O, Amid I, C = O
1622	Amid I, C = O	1430	Aromatic skeletal combined with C–H
1436	Aromatic skeletal combined with C–H	1379	C-H, C-N
1258	Amid III	1154	C-O
1050	C-O	1026	Amino acid, C-H, C-C
703	C-H, C-CL	859	C-H, C-CL
		764	C-H, C-CL
		613	C-Br

Effect of dietary treatments on growth performance is shown in Table 3. During the starter period, all treatments improved (*p* < 0.05) the body weight, weight gain and FCR of birds, when compared to the control. The same results were found at the grower phase and overall the feeding period, but with more significant (*p* < 0.05) effect on body weight and weight gain in case of ginger and nano-ginger groups. Overall the feeding trial, there were no differences (*p* > 0.05) in performance parameters between the phytogenic feed additives with its nano- particles and the used antibiotic. Moreover, the mortality rate did not differ (*p* > 0.05) among the different treatments.

**Table 3:** Performance characteristics of broiler chickens in the different experimental groups

Parameters	Group						*p*- value
	Control	Antibiotic	Thyme	Ginger	Nano-Thyme	Nano-Ginger	
**Starter phase (0–21 day)**							
Live weight(g)	706.7 ± 1.67^a^	788.6 ± 5.72^b^	779.9 ± 6.58^b^	767.0 ± 10.4^b^	784.2 ± 6.64^b^	773.9 ± 6.42^b^	< 0.001
Weight gain(g)	661.1 ± 1.80^a^	743.0 ± 5.50^b^	734.3 ± 6.70^b^	721.3 ± 10.15^b^	738.6 ± 6.47^b^	728.3 ± 6.18^b^	< 0.001
Feed intake(g)	725.0 ± 4.12^a^	784.6 ± 2.22^c^	774.5 ± 0.80^c^	759.2 ± 1.30^b^	759.2 ± 1.40^b^	757.4 ± 7.60^b^	< 0.001
FCR	1.10 ± 0.00^b^	1.06 ± 0.01^a^	1.05 ± 0.01^a^	1.05 ± 0.01^a^	1.03 ± 0.01^a^	1.04 ± 0.01^a^	0.008
Mortality rate (%)	0	4.45 ± 2.22	4.45 ± 2.22	2.22 ± 2.22	2.22 ± 2.22	2.22 ± 2.22	0.65
Feed cost of production (L.E./kg)	14.96 ± 0.05^bc^	16.01 ± 0.17^d^	15.33 ± 0.13^c^	15.33 ± 0.21^c^	14.37 ± 0.28^a^	14.51 ± 0.16^ab^	< 0.001
**Grower phase (22–35 day)**							
Live weight(g)	1918.5 ± 2.06^a^	2063.2 ± 5.73^bc^	2044.4 ± 11.9^b^	2082.1 ± 4.40^c^	2047.0 ± 6.27^b^	2066.2 ± 8.55^bc^	< 0.001
Weight gain(g)	1211.8 ± 0.38^a^	1274.6 ± 11.44^b^	1264.5 ± 6.55^b^	1315.1 ± 5.96^c^	1262.8 ± 7.75^b^	1292.3 ± 15.0^bc^	< 0.001
Feed intake(g)	2169.1 ± 28.3	2094.3 ± 5.54	2086.6 ± 30.1	2095.2 ± 20.4	2071.2 ± 28.8	2132.0 ± 15.03	0.58
FCR	1.79 ± 0.02^b^	1.64 ± 0.02^a^	1.65 ± 0.01^a^	1.59 ± 0.02^a^	1.64 ± 0.02^a^	1.65 ± 0.01^a^	< 0.001
Mortality rate (%)	8.89 ± 2.22	4.60 ± 2.31	4.60 ± 2.31	6.83 ± 0.16	6.83 ± 0.16	4.45 ± 2.22	0.49
Feed cost of production (L.E./kg)	24.17 ± 0.31^b^	24.60 ± 0.26^b^	23.93 ± 0.22^b^	23.06 ± 0.30^a^	22.71 ± 0.24^a^	22.85 ± 0.20^a^	0.001
**The overall period (0–35 day)**							
Live weight(g)	1918.5 ± 2.06^a^	2063.2 ± 5.73^bc^	2044 ± 11.9^b^	2082.1 ± 4.40^c^	2047.0 ± 6.27^b^	2066.2 ± 8.55^bc^	< 0.001
Weight gain(g)	1872.9 ± 2.19^a^	2017.6 ± 5.94^bc^	1998.8 ± 12.0^b^	2036.5 ± 4.19^c^	2001.4 ± 6.23^b^	2020.6 ± 8.79^bc^	< 0.001
Feed intake(g)	2805.2 ± 67.3	2909.2 ± 29.8	2827.3 ± 24.8	2854.4 ± 21.5	2830.3 ± 29.0	2889.4 ± 17.8	0.35
FCR	1.50 ± 0.04^b^	1.44 ± 0.02^a^	1.42 ± 0.01^a^	1.40 ± 0.01^a^	1.41 ± 0.01^a^	1.43 ± 0.01^a^	0.02
Mortality rate (%)	8.89 ± 2.22	8.89 ± 2.22	8.89 ± 2.22	8.89 ± 2.22	8.89 ± 2.22	6.67 ± 0.00	0.96
Feed cost of production (L.E./kg)	20.86 ± 0.48^b^	21.44 ± 0.16^c^	20.79 ± 0.12^b^	20.36 ± 0.12^b^	19.62 ± 0.14^a^	19.84 ± 0.09^ab^	< 0.001

^a, b,….^Means within the same row with different superscripts are significantly different (*P* < 0.05)

“L.E./kg” means livre égyptienne or Egyptian Pound per kilogram gain

Concerning the feed cost of production, the antibiotic treatment had the highest cost (*p* < 0.05) at the starter period, but the ginger, nano-thyme, and nano-ginger groups had the lowest (*p* < 0.05) production cost at the grower phase. During the overall period, the feed cost of production was the highest in antibiotic group, but it was the lowest in ginger and nan-ginger treatments.

There was no effect of dietary treatments on carcass yield or relative organs weight of broilers, except bursa of Fabricius and visible fat (Table 4). It was observed that the phytogenic feed additives and their nano-compounds increased (*p* < 0.05) the weight of bursa, but the antibiotic treatment produced an effect similar (*p* > 0.05) to the control. Moreover, no significant difference was noted in the weight of spleen by feeding of thyme, ginger and their nano particles. The visible fat % was the highest in antibiotic treatment, but was the lowest in ginger and nano-ginger groups.

**Table 4:** Carcass and organs weight relative to body weight (%) of the different groups at the end of the experiment (Mean ± SE)

Character *	Group						*p*- value
	Control	Antibiotic	Thyme	Ginger	Nano-Thyme	Nano-Ginger	
Dressing value	71.74 ± 0.83	71.79 ± 0.98	72.08 ± 0.81	70.28 ± 0.77	72.43 ± 0.84	70.56 ± 0.94	0.47
Liver	1.92 ± 0.12	2.04 ± 0.02	2.35 ± 0.31	2.38 ± 0.25	2.30 ± 0.14	2.25 ± 0.21	0.54
Gizzard and proventriculus	2.50 ± 0.05	2.65 ± 0.21	2.33 ± 0.24	2.55 ± 0.14	2.35 ± 0.16	2.38 ± 0.12	0.69
Heart	0.49 ± 0.07	0.42 ± 0.04	0.46 ± 0.05	0.47 ± 0.03	0.40 ± 0.06	0.40 ± 0.05	0.47
Spleen	0.17 ± 0.02	0.15 ± 0.01	0.21 ± 0.03	0.21 ± 0.03	0.18 ± 0.03	0.20 ± 0.03	0.48
Bursa of Fabricius	0.14 ± 0.02^b^	0.12 ± 0.02^b^	0.23 ± 0.01^a^	0.20 ± 0.02^a^	0.18 ± 0.01^a^	0.18 ± 0.02^a^	0.01
Visible fat**	1.91 ± 0.07^b^	2.11 ± 0.07^c^	1.85 ± 0.03^b^	1.58 ± 0.05^a^	1.88 ± 0.07^b^	1.62 ± 0.03^a^	< 0.001

* Calculated as a percentage of the live body weight before slaughtering at the end of the experiment

** It is the fat found subcutaneously and around the viscera

^a, b,….^Means within the same row with different superscripts are significantly different (*P* < 0.05)

Regarding the meat quality, the chemical composition of breast muscle was not affected (*p* > 0.05) by the dietary treatments (Table 5). Also, the pH values, water holding capacity and shear force was similar (*p* > 0.05) among the treatments. Moreover, there were significant differences (*p* < 0.05) between the treatments on breast colour. The dietary supplements increased the brightness (L*) and reduced the redness (a*) of breast meat. The yellowness (b*) of meat was increased by antibiotic, thyme and ginger, but was similar to the control in case of nano thyme and nano ginger groups. The sensory properties of breast meat did not (*p* > 0.05) differ among the treatments except flavors. The flavor values were increased (*p* < 0.05) in phytogenic additives and their nano-particles than in antibiotic and control treatments. Moreover, the tested treatments did not (*p* > 0.05) affect the chemical composition and pH values of thigh muscle (Table 6). However, the water holding capacity was increased (*p* < 0.05) in thyme and ginger, and their nano- products. These herbal additives with their nano-particles improved (*p* < 0.05) the flavor of thigh meat, but other sensory traits were not influenced (*p* > 0.05) by the different treatments.

**Table 5:** Physiochemical and sensory characteristics of broiler chicken breast muscle in the different experimental groups at the end of experiment (Mean ± SE)

Item	Group						
	Control	Antibiotic	Thyme	Ginger	Nano-Thyme	Nano-Ginger	*p*- value
**Chemical composition %**							
Moisture	71.34 ± 0.89	73.41 ± 1.26	72.74 ± 1.15	71.41 ± 0.99	72.25 ± 0.89	71.22 ± 0.63	0.56
Crude protein	84.78 ± 1.00	83.65 ± 0.55	84.70 ± 0.71	84.62 ± 0.27	84.78 ± 0.49	84.48 ± 0.96	0.86
Ether extract	10.63 ± 0.88	11.91 ± 0.64	10.77 ± 0.68	10.24 ± 0.36	10.63 ± 0.73	10.35 ± 0.64	0.58
Ash	2.39 ± 0.32	1.92 ± 0.08	2.11 ± 0.42	2.14 ± 0.39	2.49 ± 0.29	2.30 ± 0.30	0.82
**Physical characteristics**							
pH at 2 h	6.20 ± 0.09	6.27 ± 0.01	6.13 ± 0.04	6.35 ± 0.14	6.07 ± 0.03	6.09 ± 0.05	0.15
pH at 24 h	5.6 ± 0.07	5.67 ± 0.01	5.55 ± 0.04	5.75 ± 0.13	5.50 ± 0.02	5.52 ± 0.05	0.14
Water holding capacity %	68.43 ± 5.48	68.27 ± 5.09	75.97 ± 5.80	75.17 ± 5.14	71.63 ± 1.67	78.30 ± 1.82	0.54
Shear force	2.40 ± 0.11	3.26 ± 0.23	2.94 ± 0.16	3.36 ± 0.39	3.27 ± 0.26	3.17 ± 0.22	0.09
*Hunter color*							
Brightness (L*)	56.72 ± 0.08^c^	58.06 ± 0.07^b^	57.41 ± 0.01^b^	60.73 ± 0.19^a^	60.40 ± 0.20^a^	59.43 ± 0.05^a^	< 0.0001
Redness (a*)	11.90 ± 0.17 ^a^	8.47 ± 0.015^c^	9.97 ± 0.00^b^	8.29 ± 0.15^b^	7.77 ± 0.06^c^	9.00 ± 0.03^b^	< 0.0001
Yellowness(b*)	11.30 ± 0.18^ac^	13.07 ± 0.05^b^	12.71 ± 0.14^b^	13.20 ± 0.22^b^	10.69 ± 0.09^a^	11.39 ± 0.04^c^	< 0.0001
**Sensory attributes**							
Appearance	8.63 ± 0.01 ^a^	8.68 ± 0.04	8.62 ± 0.01	8.53 ± 0.03	8.62 ± 0.05	8.57 ± 0.04	0.16
Flavor	8.77 ± 0.02 ^a^	8.76 ± 0.02 ^a^	9 ± 0.00 ^b^	8.95 ± 0.03 ^b^	8.93 ± 0.04 ^b^	8.94 ± 0.035 ^b^	< 0.0001
Tenderness	8. 62 ± 0.01	8.59 ± 0.01	8.61 ± 0.01	8.56 ± 0.02	8.58 ± 0.02	8.59 ± 0.01	0.09
Overall acceptability	8.66 ± 0.003	8.67 ± 0.01	8.74 ± 0.01	8.68 ± 0.02	8.71 ± 0.03	8.70 ± 0.03	0.12

^a, b,….^Means within the same row with different superscripts are significantly different (*P* < 0.05)

**Table 6:** Physiochemical and sensory characteristics of broiler chicken thigh muscle in the different experimental groups at the end of experiment (Mean ± SE)

Item	Group						
	Control	Antibiotic	Thyme	Ginger	Nano-Thyme	Nano-Ginger	*p*- value
**Chemical composition %**							
Moisture	70.02 ± 0.92	71.51 ± 0.69	70.25 ± 0.66	70.78 ± 0.94	70.68 ± 0.31	70.17 ± 0.71	0.73
Crude protein	78.87 ± 0.52	79.03 ± 0.55	79.28 ± 0.60	80.37 ± 0.66	79.26 ± 0.81	79.88 ± 0.59	0.57
Ether extract	14.81 ± 0.52	15.11 ± 0.44	14.02 ± 0.49	13.27 ± 0.62	14.59 ± 0.59	13.85 ± 0.51	0.27
Ash	3.06 ± 0.03	3.14 ± 0.09	3.21 ± 0.11	3.21 ± 0.03	3.09 ± 0.05	3.14 ± 0.02	0.49
**Physical characteristics**							
pH at 2 h	6.42 ± 0.05	6.33 ± 0.01	6.24 ± 0.04	6.34 ± 0.05	6.21 ± 0.06	6.27 ± 0.04	0.08
pH at 24 h	5.81 ± 0.04	5.74 ± 0.01	5.65 ± 0.04	5.75 ± 0.05	5.63 ± 0.05	5.68 ± 0.04	0.07
Water holding capacity %	79.13 ± 2.89^b^	79.47 ± 2.73^b^	88.63 ± 1.54^a^	86.13 ± 3.40^a^	91.23 ± 2.34^a^	86.53 ± 3.87^a^	0.008
**Sensory attributes**							
Appearance	8.69 ± 0.12	8.66 ± 0.10	8.63 ± 0.10	8.51 ± 0.10	8.48 ± 0.03	8.58 ± 0.11	0.55
Flavor	8.69 ± 0.01 ^a^	8.77 ± 0.003 ^a^	8.9 ± 0.003 ^b^	8.89 ± 0.003 ^b^	8.89 ± 0.01 ^b^	8.84 ± 0.03 ^b^	< 0.0001
Tenderness	8. 80 ± 0.10	8.82 ± 0.10	8.72 ± 0.01	8.72 ± 0.01	8.81 ± 0.003	8.77 ± 0.05	0.69
Overall acceptability	8.73 ± 0.04	8.75 ± 0.05	8.75 ± 0.03	8.71 ± 0.03	8.73 ± 0.01	8.73 ± 0.01	0.92

^a, b,….^Means within the same row with different superscripts are significantly different (*P* < 0.05)

The dietary treatments reduced the total *Enterobacteriaceae* count in the intestine of birds (Table 7). However, no significant (*p* > 0.05) effect of dietary treatments on coliforms count was observed. Moreover, these treatments reduced (*p* < 0.05) the total aerobic mesophilic count of breast and thigh meat when compared to the control. The effect of phytogenic additives and their nano-particles on bacterial count in the intestine or meat was similar to that of antibiotic. Nevertheless, the reduced effect of nano-thyme on the bacterial count of thigh was greater than in other treatments.

**Table 7:** Bacterial count (log cfu/g) of intestine and meat of broiler chicken in the different experimental groups at the end of experiment (Mean ± SE)

Item	Segment	Group						*P*- value
		Control	Antibiotic	Thyme	Ginger	Nano-thyme	Nano-ginger	
Enterobacteriaceae count	Intestine	5.67 ± 0.11^a^	5.25 ± 0.09^ab^	5.30 ± 0.06^ac^	4.59 ± 0.17^b^	4.78 ± 0.21^bc^	4.75 ± 0.17^bc^	0.0014
Coliforms		3.15 **±** 0.19	2.95 **±** 0.20	3.06 **±** 0.18	2.98 **±** 0.13	3.01 **±** 0.17	2.96 **±** 0.15	0.152
Total aerobic mesophilic count	Breast	6.21 ± 0.16^a^	5.35 ± 0.10^b^	5.13 ± 0.06^b^	4.84 ± 0.29^b^	4.96 ± 0.10^b^	4.78 ± 0.11^b^	< 0.001
Total aerobic mesophilic count	Thigh	6.36 ± 0.18^a^	5.46 ± 0.22^b^	5.24 ± 0.01^bc^	5.39 ± 0.05^bc^	4.69 ± 0.21^c^	5.48 ± 0.076^b^	0.002

^a, b,….^Means within the same row with different superscripts are significantly different (*P* < 0.05)

## Discussion

Antibiotic feed additives have long been used as growth promoting supplements to enhance yields in poultry production [6]. However, the routine use of antibiotics in the diet of broilers is now considered to cause an increase in antimicrobial resistance of human and animal bacteria [2]. For this purpose, various compounds such as phytogenic feed additives and essential oils can be used as alternatives. Nowadays, using of nano-compounds of phytogenic ingredients in poultry feeding is of special interest, to reduce its amounts in the diets as well as to increase its bioavailability.

### Growth performance

Phytogenic feed additives have received an increased attention as possible growth performance enhancers for animals in the last decade [24]. In the present study, all treatments improved the body weight, weight gain and FCR of broilers, when compared to the control, but with more significant effect on body weight and weight gain in case of ginger and nano-ginger groups. The beneficial effect of thyme on productive performance can be explained on the basis that stimulatory effect of essential oils and related substances derived from thyme on growth and digestion [7]. The major derived compounds of thyme are thymol and carvacrol. These composites used for the appetizing, stimulating effect on digestion and exhibit beneficial effects in poultry health and production [6]. Lee et al. [25] found that essential oils of thyme had a stimulating effect on the animal digestive system, due to the increase of digestive enzymes and improve nutrients utilization through the enhanced liver function. Volatile oils from thyme were evaluated as inhibitors of microbial development [26]. Moreover, thymol has found to reduce the number of coliforms within the intestinal digesta of chickens [27]. Herb derivatives may have an effect through an increase in production of lactic acid bacteria, therefore increasing the number of beneficial bacteria and decreasing the presence of gram-negative bacteria [27]. The effects of thymol and carvacrol on poultry performance could be attributed to increased efficiency of feed utilization [25]. Recently, Hassan and Awad [28] reported that using of thyme in broiler diets had significant positive effect on performance and immunity indices. Naderiboroojerdi et al. [6] found that thyme improved the feed conversion rate and body weight, but not affect the feed intake. However, other studies Cross et al.; Sadeghi et al. [27, 29] reported that the addition of thyme had no significant effect on poultry feed conversion ratio.

Ginger (*Zingiber officinale*) is commonly used as a food spice and as a herbal medication [11]. The main important composites in ginger are gingerol, gingerdiol and gingerdione, which have the ability to enhance digestive enzymes secretion, decrease the microbial action and having anti-oxidative activity [12]. Kairalla et al. [30] reported that the increase in body weight gain and feed conversion efficiency in birds fed 1.0 and 1.5% of ginger powder could be due to the fact that herbal plant may provide some compounds that improve the digestion and absorption of dietary nutrients. The obtained results were in line with the finding of Demir et al. [5] who observed an increase in the body weight by using ginger in broiler diets. Some research found that red ginger can be used as stimulant for feed digestion and conversion, resulting in an improved body weight gain. Moreover, Herawati and Marjuki [31] noticed that birds fed diets containing ginger up to 2% had better feed conversion rate than un-supplemented ones. The positive effect of ginger in broiler diets on the body weight, weight gain and feed conversion ratio can be explained by the fact that ginger have medical and chemical properties which are responsible for taste and increased the digestive enzymes production [32]. Moreover, ginger has antibacterial and anti-inflammatory activities. Also, the active ingredients in ginger resulted in formation of more stable intestinal flora and improved feed conversion efficiency in consequence of better digestion [32]. However, using large concentrations of herbal plant directly in diets affects their sensory qualities and may reduce feed intake due to poorer palatability [27]. Nano preparations can result in improved feed flavour, easier handling, less used amounts of ingredients, increased stability, delayed essential oils release in the digestive tract, and increased bioavailability [33]. There is a scarcity of information on the nano phytobiotic in broiler feeding. Hosseini and Meimandipour; Nouri [34, 35] found that nano thyme enhanced broiler performance through increasing body weight gain and FCR at different growing periods. The use of nanotechnology is recently proposed as a gold standard for the preparation of phytobiotics. Nanotechnology could provide protection against various harsh condition as well as enhancement of solubility and bioactivity [36, 37]. Moreover, the inclusion of nano-phytobiotics in broiler chickens diet also reported to have higher growth promoting effects as compared to the free-phytobiotics [35, 38].

### Carcass characteristics

The dressing value and the relative weight of the liver, gizzard and proventriculus, heart, and spleen showed no significant differences in all different treatments. Our results agree with [9, 39, 40] who found that the inclusion of ginger in broiler chicken diet had no marked effect on dressing percentage and the relative weight of internal organs. Nano thyme did not affect the carcass yield and the relative weight of internal organs (heart, gizzard, liver), as reported by [41]. It has been noticed that the feeding of thyme, ginger and their nano particles stimulated the growth of Fabricius bursa and spleen of broiler chickens and caused an increase in their weight. In this respect, Manafi [40] found that the bursa of Fabricius’ relative weights decreased significantly in BMD-treated broiler chicken groups. Percentages of immunity organs (bursae and thymus) as indicators of immune situation were improved in the treated groups than the control one, as described by [42]. The presence of bioactive compounds in thyme and ginger probably stimulates cell proliferation in these organs, thus improved the condition of the bird’s immune system. Perhaps the higher relative weight in the bursa of Fabricius indicates the effect of thyme, ginger and their nano inclusions on the bird’s immune status [43].

For the visible fat %, ginger (10 g/kg) and nano-ginger (1 g/kg) fed groups showed significantly lower values than the control and other treated groups. On the other hand, the antibiotic-raised group (0.5 g/kg) had the highest visible fat percentage. Several studies showed that the addition of ginger and its essential oils to the broiler chicken diets as growth promoters significantly reduced the abdominal fat [44–46]. The reduction in the percentage of abdominal fat in broiler chicken fed on diets supplemented with ginger powder may be attributed to phenolic compounds, mainly gingerenone A, 6-shogaol, and 6-gingerol which inhibit endogenous fatty acids synthesis and enhance fatty acid catabolism [47], resulting in a low-fat deposition in broiler meats.

### Meat quality parameters

Meat quality describes the overall meat characteristics such as its physical, chemical, microbiological, sensory, technological, hygienic, nutritional and culinary properties [48]. The physico-chemical quality of meat including, pH, water holding capacity, color, tenderness, chemical composition, etc. is very important and strongly affects the consumer acceptability of such meat.

The pH of broiler chicken meat is considered a valuable parameter for evaluating its quality as it has a direct bearing on tenderness, water-holding capacity, colour, juiciness and shelf life [49, 50]. Moreover, no significance differences were observed in the pH values of broiler chicken breast and thigh at 2 and 24 h post-mortem among the studied groups. Similarly, Gumus and Gelen [51] found that the dietary supplementation with thyme essential oil did not affect the pH value of breast and drumstick meat. Feeding of broiler chicken on ginger-supplemented diet slightly increased the pH of its meat [31]. The ultimate pH_24_ of the control and treated groups were 5.5–5.8 at which the specific flavor and taste of meat are formed as comparable to [52].

Water holding capacity (WHC) is one of the most important functional properties of raw meat and is directly related to its color and tenderness [48]. The inclusion of antibiotic, thyme, ginger, nano-thyme and nano-ginger in the broiler diets did not affect the WHC of breast and thigh meat.). However, Herawati and Marjuki [31] found that feeding of red ginger (0.5-2%) slightly decreased the WHC of broiler chicken meat.

The meat tenderness or shear force is the most important and determining factor for consumer satisfaction with poultry meat [53]. No significant differences were found in the tenderness of broiler chicken breast samples among the groups. This result is in agreement with Petrov et al. [52] who reported that the tenderness of broiler breast was not influenced by the dietary supplement type including Immunoßeta, garlic, or herbal mix (ginger, thyme, yarrow). On the contrary, the dietary supplementation of red ginger to broiler chicken slightly increased its meat tenderness [31].

The color is the most significant quality attribute of poultry meat because consumers associate it with the product’s freshness, and decide whether or not to buy the product based on its attractiveness [48]. In the current study, dietary supplementations of broiler diets with antibiotic, phytogenic additives and their nano particles had an impact on the meat color based on the results of color parameters (L, a and b). The birds fed diets supplemented with antibiotic, thyme, ginger, nano-thyme, and nano-ginger showed a tendency towards a lighter breast meat color (L) and lower redness (a) compared to the control group. In most cases, higher L* values are linked to higher oxidation, however, in some studies it was stated that possibly the original plant extract color was responsible for this result [54, 55]. Regarding *b* values (yellowness), the antibiotic, thyme, and ginger-fed groups had significantly the highest *b* values among the studied groups. No significant differences were found between both nano-thyme and nano-ginger and the control group. This result is in accordance with Sugiharto [56] who reported that phytogenic supplements may enhance pigment deposition (especially yellow pigment) in broiler chicken meats.

The chemical composition of broiler breast and thigh showed no significant differences in moisture, crude protein, ether extract, and ash contents among all the studied groups. The Ginger and nano-ginger-fed groups had the lowest ether extract values, while the antibiotic-fed group exhibited the highest ones. This result is consistent with the above-mentioned results regarding the relative visible fat percentage. In this regards, Indigofera flour and red ginger supplemented diets increased the crude protein and decreased the crude fat contents of Sensi chicken meat [57].

Regarding the sensory attributes, there were no significant differences between all treatment groups in the sensory traits (appearance, flavor, tenderness, and overall acceptability) of broiler breast and thigh meat. The dietary inclusion of thyme, ginger, nano-thyme, and nano-ginger in the broiler diets significantly improved the flavor of breast and thigh meat when compared with the control and antibiotic-fed groups. Oluwafemi et al. [58] found that the sensory evaluation of broiler chicken meat (tenderness, juiciness, flavor and aroma) was significantly influenced by garlic or ginger oil, except the meat colour which was not significantly different among the treatments. Thyme essential oil had positive influence and did not have any negative impact on the sensory attributes of poultry meat [59, 60]. The improvement in the flavor of broiler chicken meat could be attributed to the phytochemical constituents of thyme and ginger.

### Bacteriological examination

The inclusion of antibiotic, thyme, ginger, nano-thyme, and nano-ginger in broiler diets significantly reduced the total aerobic mesophilic (TAM) counts of breast and thigh meat when compared to the control. The effect of phytogenic additives and their nano particles on the TAM count was similar to that of antibiotic. The TAM count of breast and drumstick meat were found to be significantly decreased in broiler chicken received thyme essential oil supplemented diet [51]. The effect of thyme and ginger on the bacterial count could be attributed to their active constituents of broad-spectrum antimicrobial activity [61]. Moreover, the nano compounds of thyme and ginger was assumed to have the same antibacterial actions of the raw materials. One of the most important factors known to affect meat quality is the microbial load of meat, as its increase impairs the meat quality, shortens its shelf life, and poses a risk to human health [62, 63]. Although meat’s microbial load is affected by the slaughter and storage conditions, the dietary inclusion of phytogenic feed additives has a vital impact [51]. The role of the dietary inclusion of thyme and ginger in reducing TAM of chicken meat could be explained by their ability to mitigate the pathogenic intestinal bacteria (mesophilic aerobic, coliform, and Escherichia coli) and activate the beneficial ones (lactic acid bacteria), resulting in a decrease in meat contamination with intestinal content during slaughter, thus reducing the meat’s microbial load [64]. Furthermore, the antimicrobial substances found in the structure of meat are highly important for its storage without spoilage [51]. The used dietary supplements reduced the intestinal *Enterobacteriaceae* counts in broilers. The ginger supplemented diet induced significantly lower *Enterobacteriaceae* count than the thyme group. In this respect, Dehghani et al. [65] found that quail fed diets contained different levels of thyme essential oil showed lower coliforms compared to birds fed diet with antibiotic. It was reported that ginger and thyme have strong antibacterial effect against both gram-positive and gram-negative bacteria due to its phenolic compounds content [66, 67]. The mechanism of their antibacterial action involves the disruption of bacterial membrane and efflux of intracellular contents, inhibition of efflux pumps, prevention in the formation and disruption of preformed biofilms, inhibition of bacterial motility, and inhibition of membrane ATPases [61]. So, phytogenic feed additives have provided sufficient evidence to be safe and natural alternatives of antibiotic growth promoters in broiler chicken diets, to prevent microorganisms’ contamination of human food and to prevent many diseases.

## Conclusion

The obtained results indicate that thyme and ginger with their nano- particles can improve the growth performance, gut health and meat quality of broiler chicken. Moreover, these additives can be used as alternatives to antibiotic growth promoters to overcome its health hazards and the high cost of its use. The nanotechnology of herbal ingredients can reduce their amounts in poultry diets with producing the same effect of the raw materials, and this could be due to the higher bioavailability.

## Data Availability

The data of this study are available from the corresponding author upon reasonable request.
